# Harmonization for Parkinson’s Disease Multi-Dataset T1 MRI Morphometry Classification

**DOI:** 10.3390/neurosci5040042

**Published:** 2024-11-29

**Authors:** Mohammed Saqib, Silvina G. Horovitz

**Affiliations:** 1 University of Pennsylvania, Philadelphia, PA 19104, USA; msaqib3@seas.upenn.edu; 2 National Institute of Neurological Disorders and Strokes, National Institutes of Health, Bethesda, MD 20892, USA

**Keywords:** Parkinson’s disease, batch effect, classifier, magnetic resonance imaging, brain morphometry, data harmonization

## Abstract

Classification of disease and healthy volunteer cohorts provides a useful clinical alternative to traditional group statistics due to individualized, personalized predictions. Classifiers for neurodegenerative disease can be trained on structural MRI morphometry, but require large multi-scanner datasets, introducing confounding batch effects. We test ComBat, a common harmonization model, in an example application to classify subjects with Parkinson’s disease from healthy volunteers and identify common pitfalls, including data leakage. We used a multi-dataset cohort of 372 subjects (216 with Parkinson’s disease, 156 healthy volunteers) from 11 identified scanners. We extracted both FreeSurfer and the determinant of Jacobian morphometry to compare single-scanner and multi-scanner classification pipelines. We confirm the presence of batch effects by running single scanner classifiers which could achieve wildly divergent AUCs on scanner-specific datasets (mean:0.651 ± 0.144). Multi-scanner classifiers that considered neurobiological batch effects between sites could easily achieve a test AUC of 0.902, though pipelines that prevented data leakage could only achieve a test AUC of 0.550. We conclude that batch effects remain a major issue for classification problems, such that even impressive single-scanner classifiers are unlikely to generalize to multiple scanners, and that solving for batch effects in a classifier problem must avoid circularity and reporting overly optimistic results.

## 1. Introduction

Parkinson’s disease (PD) is the second most prevalent neurodegenerative disorder, afflicting up to 1% of all individuals over the age of 60 [1]. Current diagnosis relies heavily on the first appearance of motor symptoms which will is thought to occur when more than 50% of dopaminergic neurons in the substantia nigra have died [2]. Prodromal biomarkers for PD could allow for early evaluation of neuroprotective therapies with the potential of slowing neurodegeneration [2].

A T1 structural MRI (sMRI) scan is already commonly taken for PD diagnosis to rule out alternate causes for the symptoms, such as multiple system atrophy or progressive supranuclear palsy, which makes it a useful target modality for new PD biomarkers. Regional brain atrophy in PD has been reported even early on in the progression of the disease in the subcortical and basal ganglia regions of the brain [3]. Subcortical nuclei have been found to have altered shapes, with local atrophy in the left putamen and thalamus correlated with right UPDRS motor scores [4]. Different results can be driven by technical differences and even differences in the clinical population that is studied can introduce bias, thus adding to discrepancies across studies. The most robust analyses use large multicenter cohorts of thousands of patients and controls to find group differences in the structural morphometry of the brain [5]. T1 sMRI scans have the potential to find early brain structural changes in PD patients, but the small effect sizes and inconsistencies across studies hamper their use for easy identifications of early biomarkers.

To increase the sample size (and hence power) of statistical analyses involving smaller effect sizes, large datasets or multiple PD MRI datasets from different scanners can be used. Large sample sizes are necessary to sufficiently power neuroscience studies, especially when effect sizes are small [6]; however, while these statistical analyses have found reproducible group differences in sMRI scans, work on using classifiers for individuals has been limited to single-scanner or single datasets. Though statistical analyses have found group effects, adequate classifiers would be more useful for detecting an individual case of PD, as they may help with the identification of interesting biomarkers, especially for data-rich modalities such as MRI.

Prior studies have attempted machine learning approaches, such as classifiers in PD. A key source of open MRI data for many PD studies is the PPMI dataset, which consists of MRI data from multiple sites [7]. Peng 2017 used a combination of structural features as well as resting state functional MRI (rs-fMRI) to achieve an AUC of 0.84 on 69 PD and 103 HV subjects in the PPMI dataset [8]. The analysis used more than 3000 features in this cohort including correlative features, as well as a feature selection process, before running a support vector machine classifier. Though Peng used two nested cross-validations (train, valid, test folds), the analysis was not tested on another scanner.

Long et al. 2012 used both rsfMRI and voxel-based morphometry to achieve an accuracy of 0.87 in a cohort of 19 early PD, and 27 HV subjects [9]. They used a single scanner and sequence and used similar correlative feature approaches to extract more than 6000 features before using feature selection. The group used a leave-one-out cross-validation (LOOCV) approach, but did not test on another scanner, which would also hinder reproducibility on another scanner.

Salvatore et al. 2014 used only sMRI scans and achieved a sensitivity and specificity above 0.9 with 28 PD and 28 HV subjects when testing a single scanner classification task [10]. Similarly high results were achieved for a differential diagnosis task between 28 PD and 28 Pars Supranuclear Palsy (PSP) subjects, with sensitivity and specificity above 0.9. The group also used a LOOCV approach and a PCA approach on normalized brain MRI scans to test only 55 features in an SVM approach.

Huppertz et al. 2016 also considered PD classification in the context of differential diagnosis. An atlas-based volumetry study could identify PD with a sensitivity of 0.87 and specificity of 0.85 compared to PSP and MSA [11]. Huppertz indicated that PD vs. HV classification remains challenging, as brain atrophy between the two groups overlap, but found that PSP and MSA were easier due to relatively more distinct atrophy patterns.

The ENIGMA group organized the largest multi-site statistical analysis of sMRI morphometry using models where the site was a random effect [5]. While work found useful group effects only possible with such a huge sample size, individual overlap of subjects was high and effect sizes were small for early stages of PD. Of note, no classifier was tested.

MRI scanners and differing protocols can introduce significant variability into a study design. Researchers used multiple rs-fMRI datasets for determining biomarkers and finding statistically significant results and found that results found in single datasets often did not transfer to other datasets [12]. Different sMRI scanners are known to introduce additional variability to estimates of brain volume for even the same subject, due to effects such as gradient distortion [13], or different scanner or scanning protocols [14]. Of note, even differences in clinical populations between sites can introduce bias as neurobiological cofactors [15]. ComBat is the standard practice to harmonize MRI measurements across different scanners. ComBat (alternatively called NeuroComBat when applied to radiomics) handles variance as batch effects [16] and there is now significant work to extend the algorithm [17,18].

However, there is a lack of literature on the impact of NeuroComBat on classification and prediction problems, and the inherent assumptions on how any harmonization strategy is applied.

Tafuri 2022 used NeuroComBat in a LOOCV approach on a multisite PPMI dataset approach to achieve an AUC score of 0.77 using an SVM LASSO approach, which was an improvement over the 0.71 score without NeuroComBat [19]. NeuroComBat was applied before a nested CV approach, which allows for easier processing and keeps the same model between folds, but also means test set data must be known beforehand to create the NeuroComBat model.

In general, there remains insufficient study on the use of multiple sMRI datasets for PD classification. Though group effect studies are useful, we hope to study the feasibility of classification for PD. This is even though there are now various multi-center MRI protocols for the identification of biomarkers for PD [5,7]. Here, we look at the strengths and limitations of a PD sMRI classifier trained on multiple datasets at once.

We hypothesize that the scanner-specific classifier will perform well for its own scanner but will perform poorly for other scanners. We also hypothesize that scanner-specific classifiers will excel compared to a multi-scanner classifier’s scanner-specific metrics. However, after harmonization, we expect scanner-specific metrics to match or exceed single-scanner classifier metrics.

## 2. Materials and Methods

### 2.1. Datasets

We used datasets of T1 sMRI scans specifically targeted for PD. We collected three datasets at our institution [20,21,22], and also processed three public datasets. The three public datasets included the PPMI dataset [7] (See https://www.ppmi-info.org/access-data-specimens/download-data, accessed on 1 August 2021), the Neurocon dataset [12], and Tao Wu dataset [12]. Of note, the PPMI dataset itself consists of multiple scanners and sites, which we could identify with metadata. We used T1 PPMI data that was available as of August 2021. Parkinson’s disease diagnosis criterion varies according to dataset. For NIH-specific datasets, neurologists used the UK Parkinson’s Disease Society Brain Bank Clinical Diagnosis Criteria [23].

The device serial ID from DICOM file metadata uniquely identified a specific site/scanner for each scan. Multiple scans from different patients with the same scanner shared this serial number. The Neurocon and Tao Wu (See https://fcon_1000.projects.nitrc.org/indi/retro/parkinsons.html, accessed on 1 August 2021.) datasets did not include DICOM file metadata, so we assumed that the scans came from the same scanner for each dataset. We only included a device if it had at least 10 HV and 10 PD subjects. When more than one visit was available, we also used only the first scan for each subject, to avoid reusing the same subject and introducing additional bias. Our final combined cohort was 372 subjects, including 216 PDs and 156 HVs, from 11 sites, including 6 of the best-represented device serial IDs from PPMI (See Supplementary Figures S1 and S2). Images acquisition parameters are described in Supplementary Materials.

### 2.2. Terminology

In this work, we name classifiers trained on all scans from all scanners as multi-scanner classifiers. In contrast, we also look at classifiers trained specifically on a single scanner as a single-scanner classifier.

Single-scanner classifiers will not need to be harmonized during training, as they are trained on only scans from the single scanner.

For multi-scanner classifiers, we look at scanner-specific metrics to look at how a multi-scanner classifier performed on a specific scanner. We can compare a scanner-specific metric (from the performance of a multi-scanner classifier) against a single-scanner classifier’s metric to see if our approach is able to harmonize data between classifiers.

### 2.3. Data Processing

**Figure 1:**
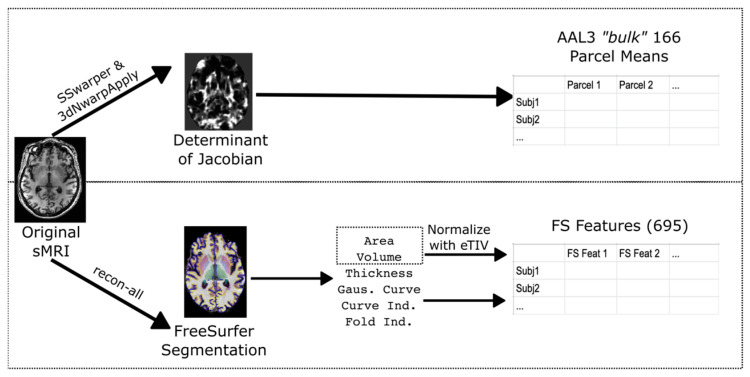
sMRI scan morphometry was calculated with two separate methods, a determinant of the Jacobian method, and FreeSurfer-based morphometry. AAL3 Determinant of Jacobian (DOJ) measures expansion/contraction for each voxel to fit a template, while FreeSurfer (FS) uses physical measurements based on constructing surfaces of the gray and white matter. Of note, some FreeSurfer values were corrected for estimated Total Intracranial Volume (eTIV) by simple division. [27] The actual eTIV is passed as a feature for FS features as well. DOJ values were subtracted by 1 to center the DOJ values at 0 (i.e., no regional increase or decrease).

Morphometry is the study of a subject brain using physical measurements of the sMRI scan. There are morphometric approaches implemented in various software pipelines [24] to capture variability in brain shape and size. We tested FreeSurfer [25] (FS) and AFNI [26] to process sMRI and extract morphometric measurements of the brain. Details of processing are shown in Supplementary Materials and Figure 1. This processing creates quantitative measures characterizing the shape of the brain.

### 2.4. NeuroComBat Algorithm

The ComBat algorithm was originally designed to solve differences in batch effects in microarray datasets [28], but is effective for neuroimaging site-effects, such as differing scanners, sequences, or acquisition procedures [15] and is renamed NeuroComBat in such cases. To harmonize between scans from different scanners, we create a NeuroComBat model with age and sex covariates and fitted to the data using an empirical Bayes algorithm provided in previously published software [16]. The NeuroComBat model is within our full pipeline, and is trained independently of the classifier, but with necessary dependency on the train, valid, and test set used for the classifier.

**Figure 2:**
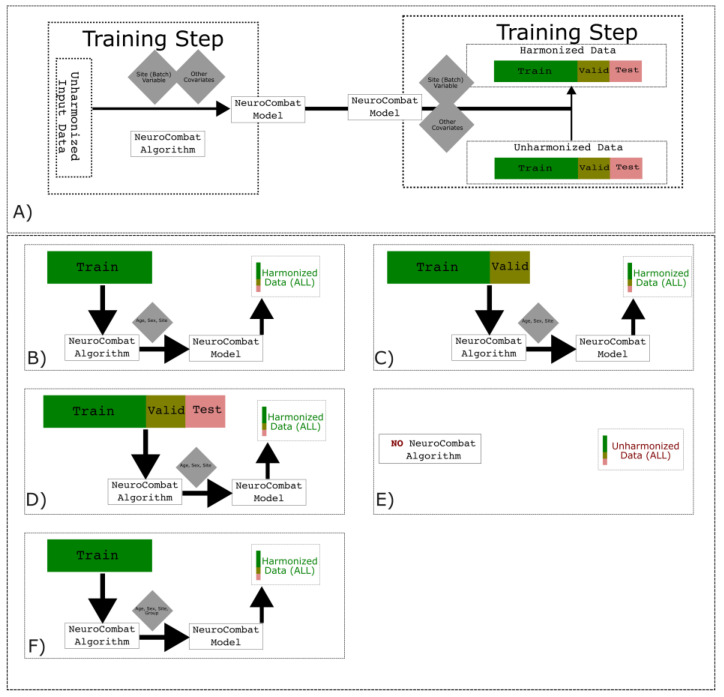
(**A**) NeuroComBat is the application of the ComBat algorithm to neurological data. The algorithm takes site as a batch effect, along with other covariates that may help explain variance from an input dataset, and is used to harmonize the full train/valid/test set. The NeuroComBat algorithm can be applied with different splits, depending on the final assumptions of the multi-scanner classifier. We test learning the NeuroComBat model on just the train set (**A**), on the train-valid set (**B**), and all the data (**C**) as a comparison and to see if a NeuroComBat model fit on only classifier-trained data are sufficient to remove site-effects such that a multi-scanner classifier. We use the model to harmonize all data in our preprocessing pipeline. We compare against not using any NeuroComBat model (**D**). Our NeuroComBat model is trained on the scanner as the site-effect variable to be removed, with age and sex covariates. As a control, we compared NeuroCombat models against simply training data without any NeuroCombat model (**E**). We additionally test a NeuroComBat model that is fit with a group covariate on a train set only, though such a model still introduces data circularity when harmonizing validation and testing data (**F**).

This model fits with data from a subset of scans from our pipeline (Figure 2), but is used to transform all scans to be harmonized. We test both with and without the NeuroComBat model to see if it is useful in the multi-scanner classifier. We include both age and sex as covariates alongside the scanner identifier to generate the model. When training scanner-specific classifiers, NeuroComBat is not used. We include both PD and HV subjects when fitting the NeuroCombat model, as ComBat algorithms require ~20 scans per scanner and should attempt to model both site-effects from the different scanners and site-effects due to differences in the disease population (i.e., neurobiological cofactors) from differing populations [15].

We do note that while the group can be included as a possible covariate for some NeuroComBat approaches, including it for a machine learning approach leads to information leaking; we would need to know the group label ahead of time to harmonize data from an unseen set. We test a NeuroComBat approach with a group covariate, regardless of where the NeuroComBat model is fit using train set data only, to confirm information leakage in our model.

As shown in Figure 2, the NeuroComBat model may be fit with different splits of the data, based on assumptions of the eventual application of a multi-set scanner. We discuss these results and assumptions later in the results and discussion, to compare if NeuroComBat is successful for a classifier.

### 2.5. Data Augmentation/Class Balancing

Imbalanced datasets may hamper the performance of a classifier, so techniques around class balancing and data augmentation have been developed [29]. Most datasets and scanners that we investigated had far more PD subjects compared to HV (See Supplementary Figure S1). In addition, some scanners had nearly twice as many subjects as others. Classifiers that are fed unbalanced data may have poor performance in correctly avoiding a positive prediction for HV subjects (specificity). Scanners that are not well-represented in the training data may also suffer. We hypothesized that balancing the data between scanners could provide additional support for data harmonization, by preventing a multi-scanner classifier from becoming biased on a single scanner.

**Figure 3:**
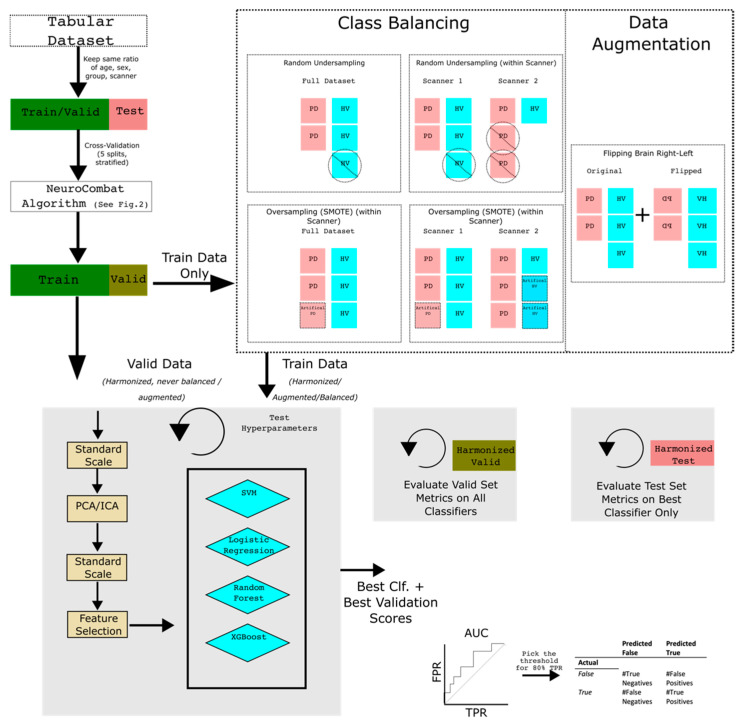
Overview of a multi-scanner classifier pipeline with the proposed selection for removing site-effects which separate between scanners and select for PD. After training and validating to find the best hyperparameters, the final metric can be taken from the test set. We use the ROC-AUC score and the balanced accuracy score as metrics to measure performance. We use a similar pipeline for the single-scanner classifier, although multi-scanner steps are removed (see Supplemental Info, Supplemental Figure S3).

As per Figure 3, we only balance and augment the training data. It is crucial to maintain the same class distribution in the validation set, so we neither augment nor balance the valid split nor do we use validation data as part of the balance and augmentation step for the training set. We test SMOTE [30] and Random Undersampling (RUS) and compared to those without balancing or augmentation.

As a hyperparameter within our hyperparameter search, we looked at whether applying a balancing strategy with regard to the scanner and group versus balancing only with regard to the group. We also tested introducing artificial scans by flipping brains left to right to increase our sample size. We tested all these options as a hyperparameter within our hyperparameter search.

### 2.6. Classifiers

We implemented classifiers using the Sklearn Python library [31] and used logistic regression (LR), random forest (RF), support vector machines (SVM), and extreme gradient boosting (XGBoost). We first stratified our datasets and set aside 20% of our dataset (71 scans) ahead of time for a test split (stratified by scanner, age, sex, and group (PD or HV)). The remaining 80% (301 scans) were used to train a classifier and validate a hyperparameter combination.

During a training step, we used 5-fold cross-validation to make train and validation splits (Figure 2) to test for the best classifier hyperparameters for multi-scanner classifiers, and a LOOCV for single-scanner classifiers. As our data have many more PD scans compared to HV, we balanced our training data and tested different methods from a library used for data augmentation [32].

We used different classifier pipelines to investigate whether we could successfully separate PD and HV subjects from cohorts collected at multiple scanners. We tested multiple hyperparameters and implemented a hyperparameter search pipeline. For each combination of class balancing, data augmentation, classifier, and datasets, we ran a random search of classifier hyperparameters to find the best classifier (See Supplemental Materials). We captured the ROC-AUC metric and used that to determine the best hyperparameter combination. To investigate whether multi-scanner classifiers were biased by the scanner, we took the scanner-specific metrics and compared them against the single-scanner classifier metrics.

### 2.7. Single Scanner Classifiers vs. Full Dataset

To compare the performance of classification between scanners, we first tested the dataset as a whole and found the multi-scanner classifier that provided the best scores without NeuroComBat. We then included NeuroComBat in a multi-scanner classifier, to repeat the full classification, and hyperparameter search procedures to evaluate whether the use of the Neuro-ComBat algorithm. We checked and confirmed if the multi-scanner performance improved.

We then compared results by limiting the training-validation split of our dataset to scans from an individual scanner. Using this device subset of the train-valid split of the data, we applied the same hyperparameter search to find the best classifier for this single scanner for each specified device. When testing with a single-scanner classifier, we could not use NeuroComBat as there was only a single site. We compared the best multi-scanner performances and generated scanner-specific metrics to compare to the single-scanner classifier.

### 2.8. Evaluation

We chose the best set of hyperparameters out of 10,000 randomly chosen hyperparameter sets using the mean 5-fold validation AUC and determined a standard deviation of the validation AUC [33].

To evaluate the results, we used AUC-ROC to characterize the performance of classifiers. We could specify the entire train-validation set and use leave-one-out (LOO) with a classifier to predict each scan when using the single-scanner classifier, as each scanner did not have enough scans. For multi-scanner classifiers, we used a 5-fold CV approach and generated error bounds for validation metrics using the folds.

For test set metrics, we only picked the best classifier from the hyperparameter search for each classifier type (SVM, RF, LR, XGBoost), dataset, and augmentation strategy and ran the predictions for the test set after training on the train-valid set. We compared test set metrics based on whether we harmonized data only using the train-valid set or with both the train-valid and test set. If the classifier can predict successfully with data harmonized only by the train-valid set, it suggests that scanner variability can be easily calculated by modeling site effects in only train-valid data. However, if the metrics work best with scanner variability characterized by both the train-valid and test set, it suggests that scanner variability cannot be easily harmonized by NeuroComBat in an unseen set.

## 3. Results

### 3.1. Subject Demographics

Datasets showed various demographic information, as shown in Table 1. Of note, PPMI datasets showed earlier stage Parkinson’s disease subjects compared to other datasets. NIH datasets showed later stage Parkinson’s disease subjects compared to other datasets. Most datasets had more PD subjects compared to HV subjects.

### 3.2. Single Scanner Classifiers vs. Full Dataset Results

As shown in Table 2, classifiers trained with the NeuroComBat model where the group was used were more performant compared to classifiers trained without; the top three validation AUCs were achieved by algorithms that modeled group effect within NeuroComBat. Both validation and test AUC were significantly higher than a classifier without the group. Even though the NeuroComBat algorithm had parameters that fit with data only from the train set, it was likely that the group, which was necessary later to harmonize the valid and test set, inflated the metrics.

We compared against algorithms that either did not use NeuroComBat or did not include the group as a covariate, as shown in Table 3. The top algorithm, by validation AUC, without the group covariate was a logistic regression classifier with a marked drop in AUC.

We tested multiple algorithms and included results in the supplemental results. (See Supplemental Table S1 and Figures S4 and S5).

Single scanner classifier results were mostly performant, except for some PPMI scanners, with an average validation score across each scanner of 0.651 ± 0.144, as shown in Table 4. Some single scanner classifiers could achieve results that approached clinical usefulness, but likely would fail to generalize to other scanners or protocols. We compared the single-scanner performance with the scores from the multi-scanner classifiers within Table 4 and found that single-scanner classifiers showed both increased and decreased metrics across a wide variety of hyperparameter choices.

Analysis for many of the results seems to indicate that augmentation strategy or balancing strategy did not matter for either the most performant algorithm that used the group covariate or the most performant algorithm that did not (see Supplemental Figure S5).

## 4. Discussion

Machine learning requires significant data to power useful analyses, but is susceptible to batch effects in unharmonized data, regardless of algorithm. This batch effect/harmonization problem is also known as a domain shift, domain adaptation problem [34], inter-scanner variability [35], and site effects [15].

Towards this end, our work applies sMRI morphometric measurements to identify potential biomarkers in multiple scanners, and multiple datasets, to identify the sources of variability. We attempt methodologies to harmonize between scanners and cohorts but find that discrepancies in classifier performance persist. To our knowledge, we are the first to attempt to look at how statistical effects for sMRI may or may not generalize, and whether classifiers can be successful with multiple datasets and scanners. We also showcase some approaches which ostensibly worked well across all scanners but may have validity issues.

We compared our work on a single scanner classifier and found that the detection of Parkinson’s varied widely depending on the scanner. The Neurocon dataset provided the best single scanner classifier metrics, perfectly predicting all scans with LOO validation; we note that LOO in such a small sample size with so many features likely indicates overfitting, and a larger sample size would likely have worse metrics. Other single scanner classifiers failed to generalize at all (i.e., PPMI scanner 1). Moreover, scanner-specific metrics from the multi-scanner classifier do not always correspond to the single-scanner classifier performance.

Our work identifies that the best models used the group variable in a NeuroComBat model to remove batch effect variability while ostensibly keeping group differences intact, leading to extremely high AUC values over 0.9. The correction of the data was conducted using a NeuroComBat model only trained on training data, but it is still likely that this approach represents some data leakage regardless; we needed to input the test label to correct data to perform prediction on an otherwise unseen test set, which defeats the purpose of a classifier.

The ComBat algorithm (or NeuroComBat, when it is applied to neuroimaging data) models site effects and produces a set of harmonized data given both a site variable and other covariables. A key issue in the batch effect problem is issues of neurobiological differences between sites, which may be due to differences in inclusion/exclusion criteria between studies [15]. Modeling neurobiological differences between sites, especially with regard to disease between sites, remains hard when the neurobiological difference is the group effect we predict. NeuroComBat may be inappropriate for classifiers, as a result, especially when the group effect is suspected to covary between sites.

We note that ComBat variants have flourished, to extend the original algorithm [15]. Though many of these variants solve use cases we did not consider, such as longitudinal changes, one interesting algorithm we did not test was modified-ComBat, which picks a specific site as a base “average” that is modified at other sites [36].

We also attempted to see if controlling for the number of scans between scanners in our multi-scanner classifier could provide better results. For example, some scanners had nearly twice as many subjects compared to other scanners, and we hypothesized that not correcting for overrepresentation could also reduce performance. Supplemental Figures S4 and S5 indicate that balancing between classes or scanners or both did not lead to drastically different metrics during the hyperparameter search.

In a previous multicenter study, statistical analyses on structural morphometry between Parkinson’s disease datasets were able to use a model similar to NeuroComBat to remove site effects and identify the progression of the disease [5]. That study used a portion of the same datasets (i.e., PPMI, Tao Wu, Neurocon) as our study and modeled group differences using the site as a random effect in a linear mixed-effects model. Robust group effects were identified, suggesting NeuroComBat and similar statistical tools may be better suited for identifying group effects rather than classification tasks. It is encouraging, regardless, that analyses of our classifiers identified that the best features for classification seemed to match early features identified in this study (see Supplemental Table S2). Features involving the entorhinal cortex were among the top five features for the multi-scanner classifier model using the group covariate in NeuroComBat, and were similarly identified as being impacted early in Parkinson’s disease. Changes in the substantia nigra DOJ were also important, especially for the multi-scanner classifier model that did not include group as a covariate.

There is no clear correspondence between UPDRS3, H&Y scores, disease duration, and classifier performance. Single scanner classifiers for PPMI scans were generally worse performing compared to other scanners. As PPMI scans were mostly within the first year of diagnosis, it is expected that little time would have passed for further degeneration, which would have made classification difficult. Many of the scans from the NIH datasets, or the Tao Wu and Neurocon datasets can be considered early PD, though they have had some further time pass since diagnosis.

PPMI uses a harmonized sequence to control for some batch effects, so the failure of a multi-scanner classifier to match single-scanner classifier models suggests further work is necessary. We note that perhaps a PPMI-specific classifier could be useful to confirm sequence harmonization can control all batch effects. Other batch effects, such as differences in scanner models or differences in the device’s magnetic fields could all contribute, despite harmonizing for sequence.

Some previous Parkinson’s disease classifier works achieved high automatic classification metrics but used a single scanner site for all scans. One study, focusing on the task of differential diagnosis using classifiers, achieved a 65.7 accuracy for detecting PD against controls, suggesting our work is competitive [11]. A review of deep learning classifiers for PD in specific cohorts found classification accuracies ranging from 95 to 100%, though many were from studies tested in smaller cohort sizes [37]. As identified in our own work, data from a single scanner with sufficiently limited cohort size may produce a classifier that has perfect metrics, but which may not hold in larger cohorts (i.e., the Neurocon dataset).

Applications of machine learning techniques (and, in particular, classification tasks) to brain diseases are increasing exponentially and many methodologies use deep learning networks [38]. Some recent work has been conducted to model batch effects using deep learning through domain adaptation methods, though more work with regard to MRI and Parkinson’s disease is necessary [34]. A new method has recently integrated NeuroComBat into a training pipeline specifically to avoid data leakage [39]. Regardless, deep learning methods should remain cautious of the same circular reasoning problems that may occur with the usage of more traditional methods to remove site effects.

Batch effects are known to have multiple sources, including scanner platform, sequence and acquisition procedures, differing pipelines, and neurobiological cofactors [15]. Though all data in this work were run with the same postprocessing pipeline, controlling for other factors remains difficult. Of note, sources of batch effects that are neurobiological cofactors, including cofactors that vary directly with the disease, remain the most difficult to control within a classifier pipeline. Parkinson’s disease can have differing diagnosis criteria, and though confirmation with a DaTScan remains among the best ways to rule out other similar diseases with Parkinsonism symptoms, we note only the PPMI datasets used this criterion [40]. Other datasets, including our internal datasets, used clinical symptoms and associated differential diagnosis to rule out other diseases. The heterogeneity of Parkinson’s disease also could act as yet another neurobiological cofactor. We note that including Parkinson’s disease status in our NeuroComBat implementation pipeline during training leads to significant improvements. Though this could be possibly due to modeling the neurobiological source of a batch effect, it also represents a very clear data leakage in the model. Machine learning analyses must avoid such circular inference to remain valid in real-world usage.

A key limitation in our work is the need for scanners with enough data. We note that many of the scanners had significantly skewed datasets, with many more PD subjects compared to HV. We necessarily limited ourselves to scanners with at least 10 HV, to properly have enough subjects to fit NeuroComBat models. Though the NeuroComBat algorithm should be resilient to small sample sizes, we found that our algorithm fluctuated wildly when including a scanner with few scans, likely due to the inability to estimate model parameters. We also note that our method requires the estimation of scanner-specific parameters ahead of time to predict a scan; completely novel scanners outside of the original training dataset cannot be handled. A further extension of our work would be to train a model to predict scanner-specific NeuroComBat model variables, similar to other works in the field [41].

Deep learning remains an exciting pathway for further exploration as well, and new methods such as Cycle-Generative Adversarial Networks (c-GANS) and Variational Auto Encoders (VAEs) [34,42]. Such networks use clever loss functions to transfer or infer images in a new site (domain adaptation) but often require significant data. Though our work was limited, a larger study could potentially use enough data to make an exploration of a deep learning framework viable. We also note that a transfer learning approach (using a previously trained model from another group) could also be a potential foundation, obviating the need for a larger dataset.

Our work finds that tweaking individual hyperparameters (choices in how we design our classification methods) does not matter as much as the initial dataset. If the initial dataset is biased by site effects, the site effects will dramatically affect classifier performance.

## 5. Conclusions

Our work was able to achieve competitive classifier metrics in small single-scanner classifiers but suggests that such metrics do not translate to larger multi-scanner datasets. Application of NeuroComBat into a classifier is challenging and requires thought and consideration to avoid overly inflating metrics due to circularity.

## Figures and Tables

**Table 1:** Demographic results show that there are similar age and sex distributions of subjects. There is a significantly lower disease duration in the PPMI cohort due to PPMI requiring an MRI scan within 6 months of diagnosis to be included in the study. N.A. means not available. UPDRS3 scores were obtained during the ON-medication state for patients in NIH and Neurocon datasets. PPMI dataset did not always specify the ON/OFF state for UPDRS3 scores.

	Group *(PD/HV)*	Age		Sex *(M/F)*		Disease Duration	H&Y Score	UPDRS3
		*PD*	*HV*	*PD*	*HV*			
NIH								
Scanner 1	24/12	62.8 ± 9.2	58.1 ± 7.4	16/8	7/5	9.4 ± 6.2	2.5 ± 0.4	17.6 ± 9.0
Scanner 2	17/19	62.2 ± 7.3	63.2 ± 6.7	12/5	15/4	6.7 ± 3.1	N.A.	28.6 ± 8.6
Scanner 3	23/20	60.4 ± 8.0	59.9 ± 7.4	12/11	9/11	7.8 ± 4.9	2.2 ± 0.5	20.6 ± 7.2
PPMI								
Scanner 1	16/12	63.1 ± 8.3	61.9 ± 12.4	12/4	10/2	0.1 ± 0.1	1.0 ± 0.6	15.2 ± 8.7
Scanner 2	22/12	62.0 ± 9.2	58.9 ± 6.9	13/9	9/3	0.1 ± 0.2	1.2 ± 0.8	15.5 ± 11.8
Scanner 3	21/13	58.5 ± 10.9	62.6 ± 9.7	13/8	9/4	0.7 ± 1.1	1.0 ± 0.9	13.4 ± 11.8
Scanner 4	14/10	61.7 ± 9.0	65.7 ± 3.1	6/8	7/3	0.3 ± 0.4	1.0 ± 0.8	15.6 ± 11.0
Scanner 5	14/11	58.7 ± 8.2	60.5 ± 8.8	11/3	7/4	0.1 ± 0.03	0.8 ± 0.8	8.6 ± 9.2
Scanner 6	19/10	63.5 ± 9.6	64.6 ± 6.8	12/7	4/6	0.3 ± 0.4	1.1 ± 0.8	12.0 ± 9.0
Other								
Neurocon	26/16	68.8 ± 10.7	67.6 ± 11.9	17/9	12/4	N.A.	1.9 ± 0.3	9.2 ± 5.3
Tao Wu	20/20	65.2 ± 4.4	64.8 ± 5.6	11/9	12/8	5.4 ± 3.9	1.9 ± 0.6	N.A.

**Table 2:** The top 3 combinations of classifier, dataset, and NC fit strategy, tested for 10 k other hyperparameters. All three of these classifiers used group to model out neurobiological batch effects.

Classifier	Dataset Used	NC Fit Strategy	Validation AUC	Test AUC
XGBoost	FS Only	Fit on Train, w/Group Covariate	0.863 ± 0.045	0.902
RF	FS Only	Fit on Train, w/Group covariate	0.841 ± 0.036	0.837
RF	Bulk + FS	Fit on Train, w/Group covariate	0.806 ± 0.056	0.866

**Table 3:** The top 3 combinations of classifier, dataset, and NC fit strategy, were tested for 10 k other hyperparameters, where group covariate is not included. As seen, the group metrics significantly fall.

Classifier	Dataset Used	NC Fit Strategy	Validation AUC	Test AUC
LR	Bulk Only	Fit on all splits, no group covariate	0.650 ± 0.052	0.550
SVC	Bulk + FS	Fit on Train, no group	0.646 ± 0.071	0.500
XGBoost	Bulk + FS	No NeuroComBat	0.644 ± 0.073	0.438

**Table 4:** Validation metrics, split up by scanner, for both single-scanner and multi-scanner classifiers. The single-scanner classifier results indicate a wide variance in performance for single-scanner performance and multi-scanner performance. Multi-scanner performance is colored green or red to indicate improvement or worsening compared to single-scanner metrics. We note NC on Train Only with Group overall has inflated AUC values, likely due to data leakage.

Scanner	Validation AUC			
	Single-Scanner Classifier	Multi-Scanner Classifier (Best)		
		No NC	NC On Train Only (No Group)	NC On Train Only (Group)
NIH Dataset 1	0.768	0.822	0.736	0.966
NIH Dataset 2	0.700	0.744	0.694	0.914
NIH Dataset 3	0.618	0.583	0.555	0.732
PPMI Scanner 1	0.486	0.614	0.643	1.0
PPMI Scanner 2	0.560	0.682	0.503	0.871
PPMI Scanner 3	0.593	0.653	0.653	0.785
PPMI Scanner 4	0.519	0.444	0.467	0.488
PPMI Scanner 5	0.573	0.518	0.527	0.873
PPMI Scanner 6	0.617	0.509	0.621	0.857
Neurocon	1.000	0.564	0.553	0.878
Tao Wu	0.727	0.645	0.719	0.930

## Data Availability

The data presented in this study are available on request from the corresponding author due to privacy and legal concerns.

## Supplementary Materials

The following supporting information can be downloaded at https://www.mdpi.com/article/10.3390/neurosci5040042/s1. Figure S1. Dataset breakdown into final processed MRI scans. Figure S2: AFNI preprocessing pipeline. Figure S3: The proposed pipeline for determining the single scanner classifier. Figure S4: The analysis of the validation scores from the hyperparameter search. Figure S5: The analysis of the validation scores from the hyperparameter search. Table S1: Classifier results for training on the full dataset. Table S2: The most important features for the classifiers.
