# Supplementary material for: Harmonization for Parkinson’s Disease Multi-Dataset T1 MRI Morphometry Classification
Source: NeuroSci. 2024 Nov 29;5(4):600–13. doi: 10.3390/neurosci5040042 (PMC11678312; doi:10.3390/neurosci5040042)
Supplement: Supplementary file 1 [file neurosci-05-00042-s001.zip › neurosci-3208452-supplementary.pdf]

Supplementary Info:

Dataset Description:

Supplemental Figure 1. Dataset breakdown into final processed MRI scans.

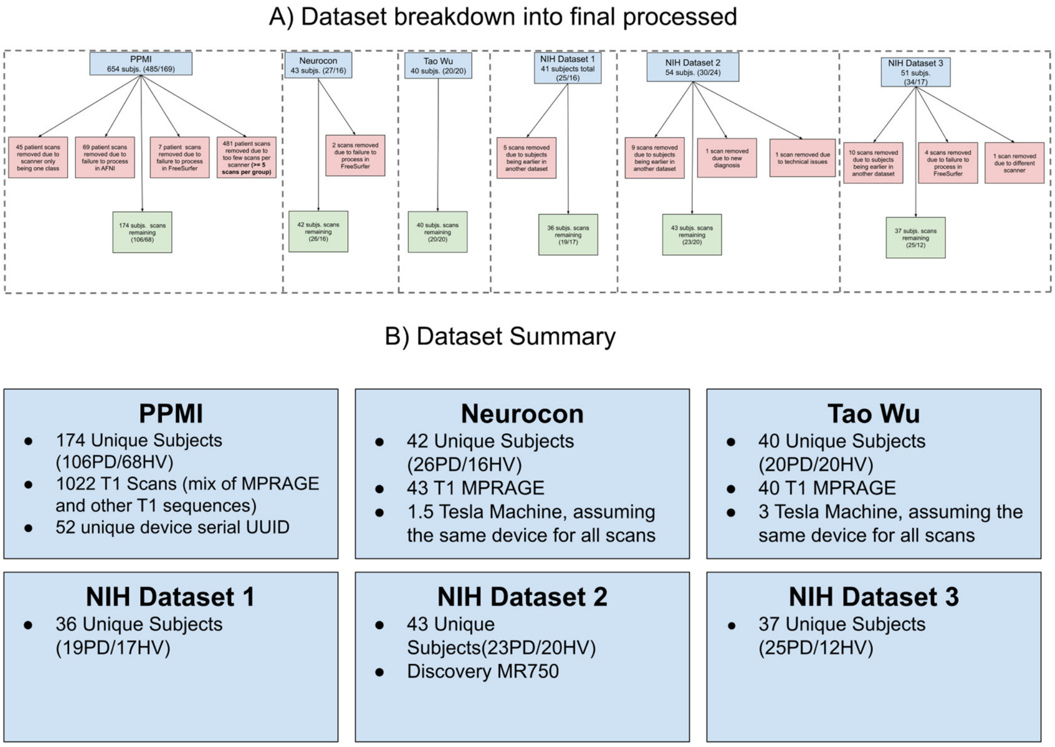

Participants:

- According to PPMI documentation, “Patients must have at least two of the following: resting tremor, bradykinesia, rigidity (must have either resting tremor or bradykinesia); OR either asymmetric resting tremor or asymmetric bradykinesia” with further confirmation based on a DaTscan.
- According to documentation provided in Badea 2017, the Neurocon cohort was diagnosed based on EFNS/MDS-ES criterion for Parkinson’s disease diagnosis.
- In documentation provided in Badea 2017, the Tao Wu dataset is described as having early to moderate stages of the disease according to the Hoehn-Yahr staging, though exact information on diagnosis is not given.
- Cases from within our Institute were diagnosed by Movement Disorders Specialists based on the UK Parkinson’s Disease Society Brain Bank Clinical Diagnosis Criteria [24].

Internal Datasets:

Our lab used internal datasets that we had gathered and ran under previous protocols. As a result, these datasets used MRI scanners at the NIH and often had overlapping subjects, as many volunteered for multiple protocols. We matched subjects by name to remove duplicate subjects between the protocols and picked the earliest scans for subjects if multiple were available to include in our analyses.

NIH Dataset 1:

3 Tesla GE Signa HDxt MRI scanner and 8-channel coil.

. MPRAGE sequence: T1-weighted anatomical images (Inversion recovery, TR: 6.536 ms, TE: 2.816 ms, TI: 450 ms, slice thickness: 1.3 mm, FoV: 240 × 240 mm, matrix size: 256 × 256) on a

NIH Dataset 2:

PD Biomarkers is a dataset to track the progression of Parkinson's Disease (PD). This dataset consists of 30 PD subjects, 24 Healthy Volunteer (HV) subjects, and 8 Prodromal subjects (EP). All subjects are tracked over 3 years, with new scans every year. 1 PD subject is excluded due to a likely alternative diagnosis. EP subjects were not included. As part of the protocol, UPDRS scores, MMSE, and MOCA scores for PD subjects, along with medications were captured. This dataset was internal to the lab. For all subjects, only first visit was included.

We used MPRAGE scans from a 3T GE DISCOVERY MR750 scanner. T1-weighted anatomical images: Magnetization prepared rapid acquisition gradient echo (MPRAGE), 3D inversion recovery, TR: 7.664 ms, TE: 3.42 ms, TI: 425 ms, slice thickness: 1 mm, 1 × 1 mm in-plane resolution, percent phase FoV: 100, flip angle: 7, matrix size: 256 × 256.

NIH Dataset 3:

This dataset consists of 36 PD and 16 HV subjects. We used a 3T Signa HDxt scanner. MPRAGE sequence: T1-weighted anatomical images (Inversion recovery, TR: 6.536 ms, TE: 2.816 ms, TI: 450 ms, slice thickness: 1.3 mm, FoV: 240 × 240 mm, matrix size: 256 × 256)

External Datasets:

We also used public facing datasets, all of which were available online as of Dec 28, 2021.

PPMI:

PPMI is the largest online public dataset of PD subject data. We searched for MRI subjects under the PD, GenCohort PD, and Control groups on 08/04/2021 and picked the first images with T1 weighting taken for each subject. This dataset consists of 705 PD subjects and 198 HV subjects. 485 PD subjects had T1 images within the first year of diagnosis and 169 HV subjects had T1 images. After quality control, 461 PD subjects, and 156 HV subjects remained. We then attempted to find the location of the scans for each subject but failed to find it among the PPMI documentation. Instead, we used metadata from the PPMI website to obtain manufacturing models of the scanners used to scan the subjects and used the manufacturer models as proxies for the location.

PPMI uses a harmonized 3D T1 sequence. We used MPRAGE sequences for the chosen scans. The included scanners were Signa HDxt, Discovery MR750, Achieva, TrioTim, Symphony, and Signa Excite. There was a mix of 3T and 1.5T scans, all with varying scanner settings. We also found that each device serial number usually only belonged to a single manufacturing model, though a few device serial numbers showed up multiple times for what were purportedly different manufacturing models. Scans from the same device serial number generally used similar scan settings.

Information about PPMI 3T sequences can be found at https://www.ppmi-info.org/sites/default/files/docs/PPMI2.0_002_MRI_TOM_Final_v3.0_20210727.pdf and is summarized here:
3D T1-weighted sequence, collected in sagittal plane. Number of slices 192, Slice thickness 1.0mm (adjusted up to 1.2 to cover full brain), voxel size in-plane resolution: 1x1mm; phase encoding Anterior-posterior. Matrix size 256x256 (no interpolation, zero-filling or ZIP factor). FOV 256mm full required. Other parameters to be set based on manufacturer.

Information about PPMI 1.5T sequences can be found at https://www.ppmi-info.org/sites/default/files/docs/archives/PPMI-MRI-Operations-Manual-V7.pdf

3D T1-weighted MPRAGE sequence. The field of view (FOV) must. Slice thickness (mm) 1.2 (or less than 1.5mm to include the vertex, cerebellum and pons); no interslice gap; voxel size in-plane resolution: 1x1mm; Matrix 256 x 256 x 170-200. Sagittal plane. All other parameters including repetition (TR) and echo (TE) time should follow your manufacturer recommendations for a T1- weighted, 3D sequence.

Neurocon/Tao Wu

These two datasets are available online as public datasets used previously to find differences in PD vs HV subjects [12]. There are 27 PD and 16 HV subjects in the Neurocon dataset and 20 PD and 20 HV subjects in the Tao Wu dataset.
The Neurocon dataset is from a 1.5 Tesla Siemens Avanto Scanner. T1-weighted MPRAGE sequence with a repetition time of 1940 ms and an echo time of 3.08 ms. Vooxel size 0.97x0.97x1 mm
The Tao Wu dataset is from a 3T Siemens Magnetom Trio scanner, using a T1-weighted MPRAGE sequence with a repetition time ofv v 1100ms and an echo time of 3.39 ms and a resolution of 1x1x1 mm

Processing Description:

AFNI Morphometry Pipeline:

AFNI implements an alignment software using SSWarper. This software runs an affine (translation, rotation, global scaling, global shearing) and nonlinear transform. We ran the SSWarper for all sMRI subjects and aligned to the MNI152_2009_T1c template. We confirmed correct alignment for all subjects by visual inspection of the quality control files.

We extracted nonlinear morphometric features using AFNI, specifically using the nonlinear warp matrix (which is the determinant of the Jacobian of the transformation and called "bulk" within the AFNI documentation). This warp matrix is derived from transforming the original subject scan to a common MRI template space. The determinant of Jacobian transform captures nonlinear regional volume expansion and contraction of brain voxels to fit the common MNI template space, while avoiding capturing global changes. We calculated the regional

expansion or contraction of every voxel using AFNI's morphometric function to compare regional differences between subjects.

We then parceled resulting deformation matrix into the AAL3 atlas and took means of each of the 166 parcels to characterize nonlinear regional volume changes of parcels of each brain of each subject in tabular form.

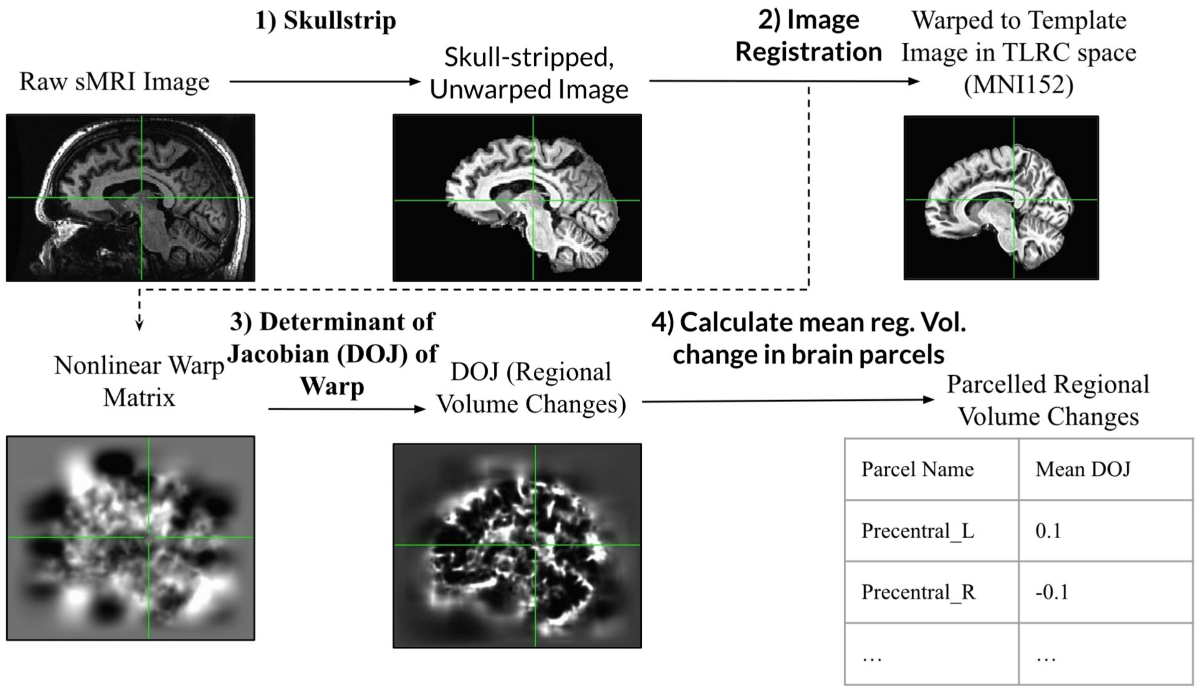

AFNI image registration to a template for a subject brain, and calculation of DOJ of the brain to quantify brain region volume changes. DOJ determines if a brain voxel had to expand or contract to fit a template space. Of note, registration is necessary in many workflows, not just DOH.

Supplemental Figure 2: AFNI preprocessing pipeline. This pipeline uses the nonlinear registration algorithm implemented in SSWarper and used alignment to MNI152 nonlinear asymmetric template to measure changes. The DOJ value is exposed as a 'bulk' value, and is subtracted by 1 to center at no regional volume change.

FreeSurfer Morphometry Pipeline:

FreeSurfer was run using the standard recon_all command. We confirmed, through visual inspection, that surfaces were correct. After FreeSurfer created surfaces and segmentations for the brain, we used the aparcstats2table and asegstats2table command to extract morphometric measurements. We used the `qatools.py` script to identify outliers and corrected subjects accordingly.

Area and volume measures from FreeSurfer were shown to vary with brain intracranial volume (eTIV), so we normalized measures by dividing by eTIV. Thickness and curvature measures did not vary with eTIV.

Initial Statistical Analyses:

Tabular Data:

We created an ANCOVA model for group with age and sex factor against each of the final morphometric measures from **Figure 1** after we had converted images into tabular data. We tested on the full dataset (train/valid and test). We use the false discovery rate for multiple comparison correction. We test with and without NeuroCombat on the values before using the t-test.

After FDR correction, only gray matter volume (normalized by the intracranial volume) is significant between groups. This feature is only significant when we use NeuroCombat beforehand to harmonize data. If we do not harmonize beforehand, no feature surves FDR correction.

Classifier Info:

Single Scanner Classifier

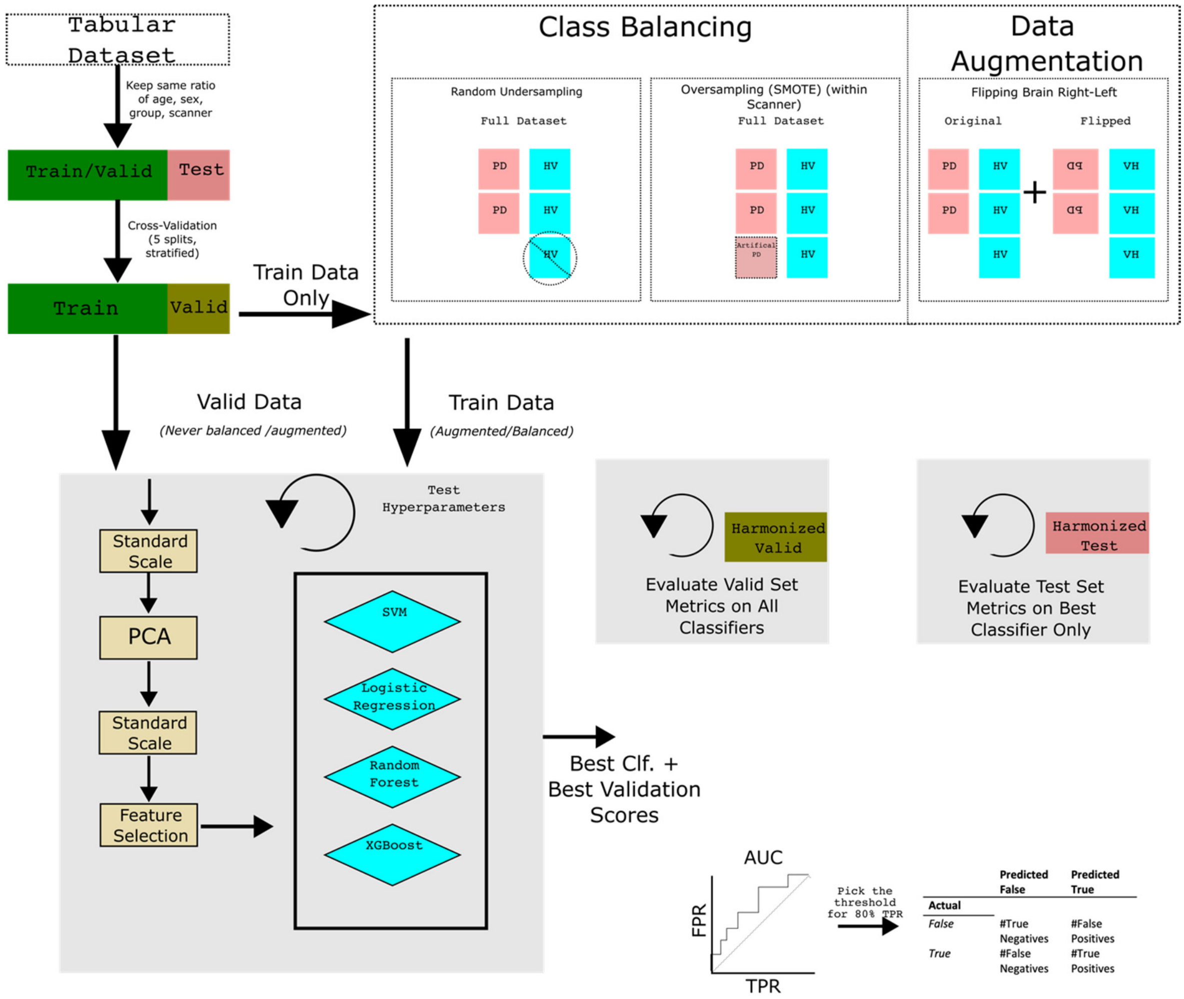

Supplemental Figure 3: The proposed pipeline for determining the single scanner classifier. As only data from a single scanner is used, we do not need to either run NeuroCombat nor do we balance number of training examples between scanners (as there is only one). We repeat this for each scanner. Training during the train-validation fold was done using LOOCV.

RandomSearch of Hyperparameters:

Hyperparameter searches can be expensive, and testing every combination of classifier hyperparameters can become computationally infeasible. For example, with the setup we used for XGBoost, we would need to test more than 400 million hyperparameter combination for a full expansive gridsearch (from the hyperparameters we tested). We would likely need to vastly reduce our search space to exhaustively find the best hyperparameter combination. Though this is a valid approach if it is known which hyperparameter is worth searching over and which isn't, we decided to keep our large search space.

To solve this, we used a random search of hyperparameters [35]. Random search can be comparable to a full search of every combination of hyperparameters, with minimal loss of metrics, for large numbers of random searches.

Randomized searching for classifiers can be modeled as a binomial distribution, with each combination of hyperparameters as a trial. To achieve a trial which has a score within the top 0.001 of all hyperparameter combinations, with 0.001 confidence, we would need to test 7000 trials. Therefore, for this paper, we test 7000 random hyperparameter combinations for every combination of classifier, harmonization strategy, augmentation strategy, and choice of morphometric measures.

Additional Results:

Full Classifier Results:

**Classifier Name**	**Uses AFNI DOJ**	**Uses FS Features**	**NeuroCombat Fit Strategy**	**Include Group as Covariate in NC**	**Validation AUC**	**Test AUC**
xgboost	FALSE	TRUE	NCstrat.TRAIN_ONLY	TRUE	0.863¬±0.045	0.903
rf	FALSE	TRUE	NCstrat.TRAIN_ONLY	TRUE	0.841¬±0.036	0.837
rf	TRUE	TRUE	NCstrat.TRAIN_ONLY	TRUE	0.806¬±0.056	0.866
xgboost	TRUE	TRUE	NCstrat.TRAIN_ONLY	TRUE	0.797¬±0.056	0.864
lr	TRUE	FALSE	NCstrat.ALL_NC	FALSE	0.650¬±0.052	0.550
svc	TRUE	TRUE	NCstrat.TRAIN_ONLY	FALSE	0.646¬±0.071	0.500

xgboost	TRUE	TRUE	NCstrat.SKIP	FALSE	0.644¬±0.073	0.438
lr	TRUE	FALSE	NCstrat.TRAIN_VALID_ONLY	FALSE	0.644¬±0.044	0.543
xgboost	TRUE	FALSE	NCstrat.TRAIN_ONLY	TRUE	0.644¬±0.032	0.528
svc	TRUE	TRUE	NCstrat.TRAIN_VALID_ONLY	FALSE	0.640¬±0.058	0.500
xgboost	FALSE	TRUE	NCstrat.TRAIN_VALID_ONLY	FALSE	0.639¬±0.026	0.438
lr	TRUE	TRUE	NCstrat.SKIP	FALSE	0.636¬±0.017	0.500
lr	TRUE	TRUE	NCstrat.ALL_NC	FALSE	0.636¬±0.057	0.497
xgboost	TRUE	FALSE	NCstrat.ALL_NC	FALSE	0.636¬±0.039	0.504
svc	TRUE	TRUE	NCstrat.TRAIN_ONLY	TRUE	0.634¬±0.074	0.506
svc	TRUE	TRUE	NCstrat.SKIP	FALSE	0.633¬±0.044	0.500
rf	TRUE	TRUE	NCstrat.SKIP	FALSE	0.630¬±0.048	0.474
svc	TRUE	FALSE	NCstrat.ALL_NC	FALSE	0.629¬±0.027	0.666
xgboost	TRUE	TRUE	NCstrat.TRAIN_ONLY	FALSE	0.629¬±0.041	0.468
rf	TRUE	FALSE	NCstrat.TRAIN_ONLY	FALSE	0.629¬±0.035	0.503
rf	TRUE	FALSE	NCstrat.SKIP	FALSE	0.629¬±0.023	0.628
xgboost	TRUE	TRUE	NCstrat.ALL_NC	FALSE	0.628¬±0.043	0.512
rf	TRUE	FALSE	NCstrat.TRAIN_VALID_ONLY	FALSE	0.626¬±0.033	0.534
lr	TRUE	TRUE	NCstrat.TRAIN_VALID_ONLY	FALSE	0.626¬±0.046	0.617
svc	FALSE	TRUE	NCstrat.TRAIN_ONLY	TRUE	0.625¬±0.026	0.500
rf	TRUE	TRUE	NCstrat.TRAIN_VALID_ONLY	FALSE	0.625¬±0.024	0.497
svc	FALSE	TRUE	NCstrat.TRAIN_ONLY	FALSE	0.624¬±0.032	0.500
xgboost	TRUE	TRUE	NCstrat.TRAIN_VALID_ONLY	FALSE	0.623¬±0.052	0.479

xgboost	FALSE	TRUE	NCstrat.SKIP	FALSE	0.619¬±0.039	0.421
xgboost	FALSE	TRUE	NCstrat.TRAIN_ONLY	FALSE	0.619¬±0.020	0.462
xgboost	TRUE	FALSE	NCstrat.SKIP	FALSE	0.619¬±0.048	0.544
rf	TRUE	FALSE	NCstrat.ALL_NC	FALSE	0.619¬±0.054	0.579
xgboost	FALSE	TRUE	NCstrat.ALL_NC	FALSE	0.618¬±0.042	0.497
svc	TRUE	FALSE	NCstrat.TRAIN_VALID_ONLY	FALSE	0.618¬±0.033	0.510
xgboost	TRUE	FALSE	NCstrat.TRAIN_ONLY	FALSE	0.616¬±0.056	0.435
lr	TRUE	FALSE	NCstrat.TRAIN_ONLY	FALSE	0.616¬±0.029	0.580
lr	FALSE	TRUE	NCstrat.TRAIN_ONLY	TRUE	0.616¬±0.045	0.526
lr	FALSE	TRUE	NCstrat.TRAIN_VALID_ONLY	FALSE	0.616¬±0.040	0.526
lr	FALSE	TRUE	NCstrat.TRAIN_ONLY	FALSE	0.615¬±0.061	0.532
rf	TRUE	TRUE	NCstrat.TRAIN_ONLY	FALSE	0.614¬±0.067	0.462
rf	FALSE	TRUE	NCstrat.SKIP	FALSE	0.613¬±0.016	0.468
lr	FALSE	TRUE	NCstrat.SKIP	FALSE	0.612¬±0.040	0.528
rf	FALSE	TRUE	NCstrat.TRAIN_ONLY	FALSE	0.612¬±0.038	0.468
lr	FALSE	TRUE	NCstrat.ALL_NC	FALSE	0.612¬±0.045	0.421
xgboost	TRUE	FALSE	NCstrat.TRAIN_VALID_ONLY	FALSE	0.612¬±0.014	0.527
svc	TRUE	FALSE	NCstrat.TRAIN_ONLY	FALSE	0.609¬±0.051	0.525
rf	TRUE	FALSE	NCstrat.TRAIN_ONLY	TRUE	0.608¬±0.031	0.509
rf	TRUE	TRUE	NCstrat.ALL_NC	FALSE	0.606¬±0.060	0.493
rf	FALSE	TRUE	NCstrat.ALL_NC	FALSE	0.600¬±0.061	0.455
svc	TRUE	TRUE	NCstrat.ALL_NC	FALSE	0.598¬±0.036	0.500

lr	TRUE	TRUE	NCstrat.TRAIN_ONLY	FALSE	0.596¬±0.060	0.469
lr	TRUE	TRUE	NCstrat.TRAIN_ONLY	TRUE	0.594¬±0.057	0.539
svc	FALSE	TRUE	NCstrat.TRAIN_VALID_ONLY	FALSE	0.593¬±0.035	0.500
rf	FALSE	TRUE	NCstrat.TRAIN_VALID_ONLY	FALSE	0.587¬±0.068	0.569
svc	FALSE	TRUE	NCstrat.SKIP	FALSE	0.586¬±0.039	0.500
lr	TRUE	FALSE	NCstrat.SKIP	FALSE	0.584¬±0.027	0.515
lr	TRUE	FALSE	NCstrat.TRAIN_ONLY	TRUE	0.583¬±0.061	0.502
svc	FALSE	TRUE	NCstrat.ALL_NC	FALSE	0.572¬±0.045	0.479
svc	TRUE	FALSE	NCstrat.SKIP	FALSE	0.571¬±0.039	0.467
svc	TRUE	FALSE	NCstrat.TRAIN_ONLY	TRUE	0.546¬±0.067	0.489

Supplemental Table 1: Classifier results for training on the full dataset. AUC metrics are for the full dataset.

Best Hyperparameters within search

Augmentation Strategy/Class Balancing Strategy

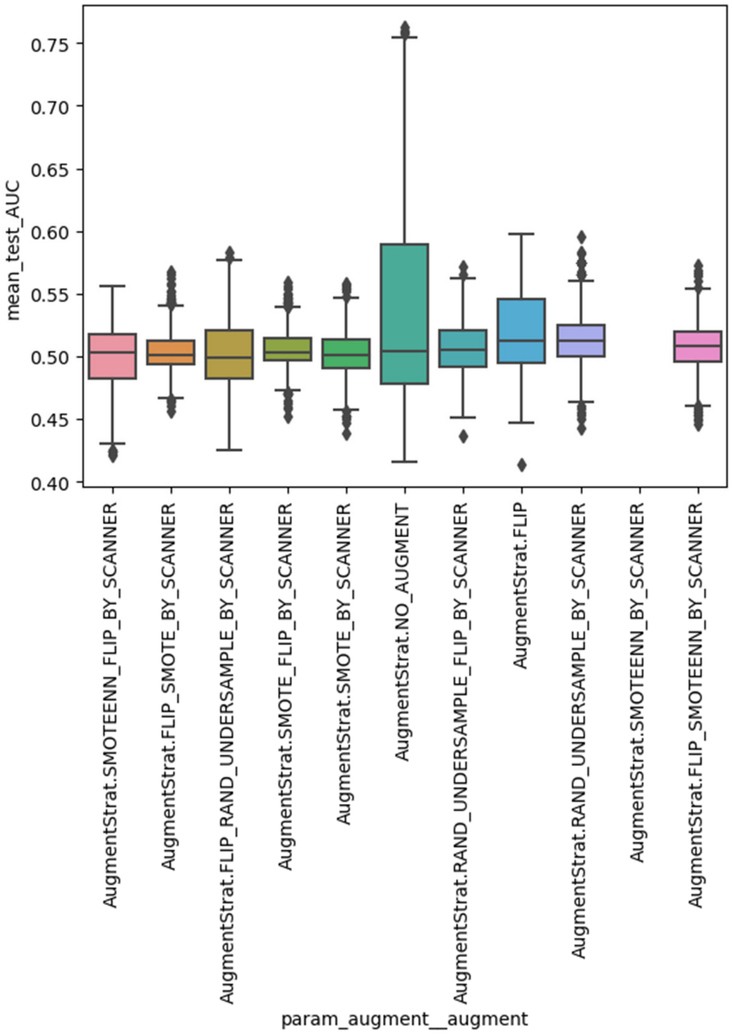

Supplementary Figure 4: The analysis of the validation scores from the hyperparameter search. The scores are plotted by augment strategy for the best classifier trained with a NeuroCombat model using a group covariate. The best strategy was neither class balancing nor augmenting data, representing the likely data leakage issue.

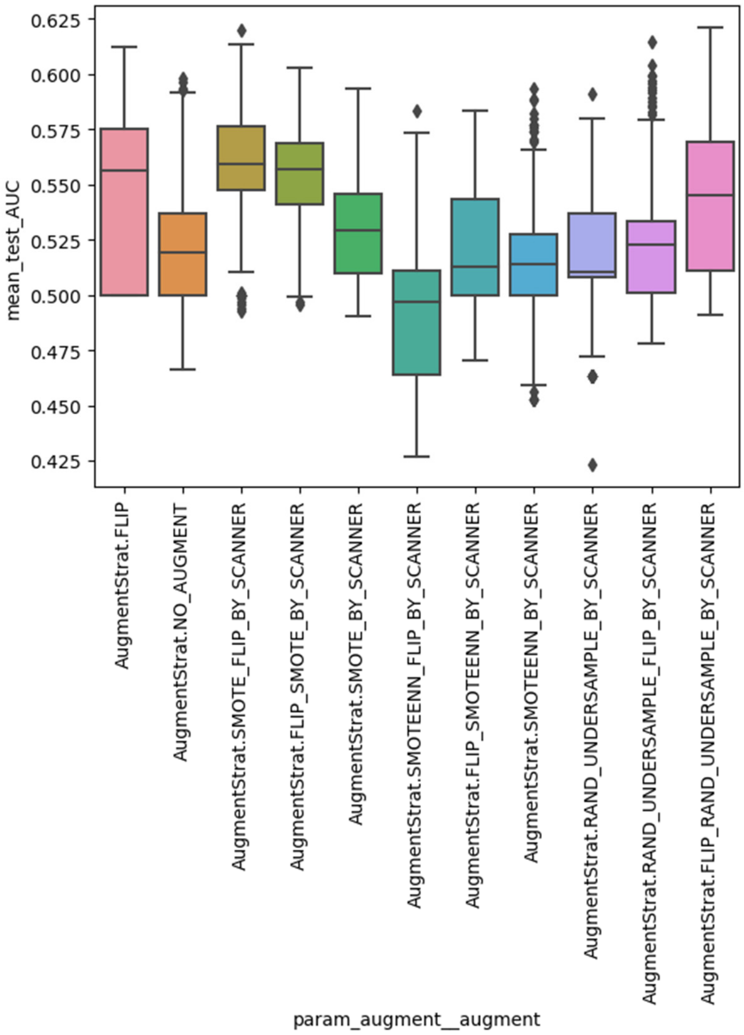

Supplementary Figure 5: The analysis of the validation scores from the hyperparameter search. Scores are plotted by augment strategy for the best classifier trained with a NeuroCombat model not using a group covariate.

Best Model With NC with Group Covariate	Best Model With NC with/out Group Covariate
Non Ventricle Supratentorial Volume	Substantia Nigra Pars Reticulum DOJ
Left Hemisphere Entorhinal Cortex Volume	Left Pallidum DOJ
Right Hemisphere Entorhinal Cortex Curvature	Right Pallidum DOJ
Right Ventral Diencephalon Volume	Ventral Thalamus DOJ
Total Gray Matter Volume	Right Olfactory Bulb DOJ

Supplemental Table 2: The most important features for the classifiers. Features from NeuroCombat preprocessing pipelines which used the group covariate and the classifier which did not use the group covariate are drastically different. Of note, when the group covariate is not

used in the NC model, the best classifier is a simpler logistic regression model trained on determinant of the Jacobian features. When the group covariate is included, the best classifier uses FreeSurfer features, and uses global features of gray matter volume, and some features, such as measures around the entorhinal cortex, that are not usually suspect in PD.
